# MendelianRandomization: an R package for performing Mendelian randomization analyses using summarized data

**DOI:** 10.1093/ije/dyx034

**Published:** 2017-04-07

**Authors:** Olena O Yavorska, Stephen Burgess

**Affiliations:** 1 Newnham College, University of Cambridge and; 2 Department of Public Health and Primary Care, University of Cambridge, Cambridge, UK

**Keywords:** Mendelian randomization, instrumental variable, causal inference, summarized data, two-sample, data parasite

## Abstract

MendelianRandomization is a software package for the R open-source software environment that performs Mendelian randomization analyses using summarized data. The core functionality is to implement the inverse-variance weighted, MR-Egger and weighted median methods for multiple genetic variants. Several options are available to the user, such as the use of robust regression, fixed- or random-effects models and the penalization of weights for genetic variants with heterogeneous causal estimates. Extensions to these methods, such as allowing for variants to be correlated, can be chosen if appropriate. Graphical commands allow summarized data to be displayed in an interactive graph, or the plotting of causal estimates from multiple methods, for comparison. Although the main method of data entry is directly by the user, there is also an option for allowing summarized data to be incorporated from the PhenoScanner database of genotype—phenotype associations. We hope to develop this feature in future versions of the package. The R software environment is available for download from [https://www.r-project.org/]. The MendelianRandomization package can be downloaded from the Comprehensive R Archive Network (CRAN) within R, or directly from [https://cran.r-project.org/web/packages/MendelianRandomization/]. Both R and the MendelianRandomization package are released under GNU General Public Licenses (GPL-2|GPL-3).

**Key Messages**

- The MendelianRandomization software package enables Mendelian randomization analyses to be undertaken using summarized data on genetic associations for a variety of previously published methods.
- The use of this package should lead to more reproducible and more credible causal inferences, due to the range of robust methods included in the package.
- However, a critical and principled approach to Mendelian randomization investigations is required for causal claims to be justified; robust methods can reveal inconsistencies in the analyses, but conclusions that rely on statistical approaches in the absence of biological understanding of the variants included in the analysis will always be speculative.

## Introduction

Mendelian randomization is the use of genetic variants as instrumental variables for assessing causal relationships from observational data.^1^^,^^2^ Increasingly, Mendelian randomization analyses are being performed using summarized data, in particular the associations (beta-coefficients and standard errors) of each genetic variant with the exposure and outcome variables.^3^ Such associations are often made publicly available by studies or consortia.^4^^,^^5^ The previously proposed inverse-variance weighted method gives the same estimates using these summarized data as the well-established two-stage least squares method that uses individual-level data.^6^^,^^7^

The inverse-variance weighted (IVW) method provides a consistent estimate of the causal effect of the exposure on the outcome when each of the genetic variants satisfies the assumptions of an instrumental variable.^6^ Two further methods have been proposed for providing consistent causal estimates from summarized data for multiple genetic variants under weaker assumptions. These are the MR-Egger method^8^ and the weighted median (or median-based) method.^9^ Several publications have used some or all of these methods,^10–12^ and the use of all three methods is recommended when there are multiple genetic variants to assess robustness of any causal finding to different sets of assumptions.^13^ Additionally, several variations on these methods have been proposed, such as the use of robust regression instead of standard linear regression in the IVW or MR-Egger methods, or the penalization of weights from genetic variants with heterogeneous causal estimates.^14^

Software code is available for the implementation of each of these methods. However, this code is currently spread among the appendices of various manuscripts. The MendelianRandomization package brings the code together in a single location and makes implementation of these methods simpler and easier to reproduce, meaning that applied researchers can focus on the most important aspects of a Mendelian randomization investigation, namely the choice of genetic variants and assessment of the instrumental variable assumptions. Code for implementing all of the methods in the paper using the Mendelian Randomization package is provided in the Online Appendix.

## Implementation and usage

The data entry function in the MendelianRandomization package is the mr_input() function. The required inputs for this function are the beta-coefficients for associations with the exposure and their standard errors, and the beta-coefficients for associations with the outcome and their standard errors. A minimal example is:

MRdata_HDL_CHD <- mr_input(bx = hdlc, bxse = hdlcse, by = chdlodds, byse = chdloddsse)

The variables hdlc, hdlcse, chdlodds and chdloddsse are provided as part of the MendelianRandomization package as example data, and represent the associations of 28 variants with high-density lipoprotein cholesterol (HDL-c) and their standard errors (hdlc and hdlcse), and the associations of the same 28 variants with coronary heart disease (CHD) risk and their standard errors (chdlodds and chdloddsse), taken from Waterworth et al.^15^ The mr_input() function ensures that the data are formatted correctly as an MRInput object for processing by one of the estimation or graphical functions. We note that the mr_input() function makes no attempt to align the genetic associations to the same effect allele; this step is critically important, but is left to the user. Genetic associations obtained from PhenoScanner are automatically aligned to the same effect allele.^16^ Special care should be taken when it is not clear whether alleles are given for the forward or backward strand, particularly for palindromic variants (C/G or A/T polymorphisms).

The IVW, MR-Egger and weighted median methods are then implemented by taking the MRInput object as an input, and outputting causal estimates and related statistics. For the IVW method, an example is:

mr_ivw(MRdata_HDL_CHD)

where MRdata_HDL_CHD is as defined above. By default, the mr_ivw() function reports the causal estimate and its standard error, a 95% confidence interval based on a normal approximation, and the residual standard error and associated heterogeneity test statistic, indicating whether the causal estimates from the individual genetic variants are similar to each other or not.^17^

Similar output is provided by the mr_egger() and mr_median() functions, except that the MR-Egger method additionally reports an intercept parameter, representing the average pleiotropic effect of a genetic variant.^8^ Several options are available to the user in the ability: to use robust regression instead of standard linear regression in the IVW and MR-Egger methods;^14^ to penalize the contribution to the analysis of genetic variants with heterogeneous causal estimates;^14^ to use an unweighted rather than a weighted analysis in the median-based method;^9^ to make inferences based on a t-distribution rather than a normal distribution; and to report a confidence interval at a different alpha level (for example, a 99% confidence interval). A maximum likelihood method mr_maxlik() that fits a likelihood model to the summarized data, allowing for uncertainty in genetic associations with both the exposure and the outcome,^6^ is also implemented in the package.

A further option is the possibility of accounting for correlation between genetic variants in either the IVW or the MR-Egger method. This illustrates one benefit of the package–although the use of generalized weighted linear regression has been proposed for performing the IVW method with correlated variants,^18^ it has not been formally proposed for the MR-Egger method,despite the extension being relatively straightforward. The package means that if a methodological advance is made for one method, it can be implemented for multiple methods, and if two methodological advances are made that can interact, their interaction can be explored by setting both options in the package.

The mr_allmethods() function can be used to implement several of the methods offered in this package at once. An example is:

mr_allmethods(MRdata_HDL_CHD, method = ”main”)

**Figure 1:**
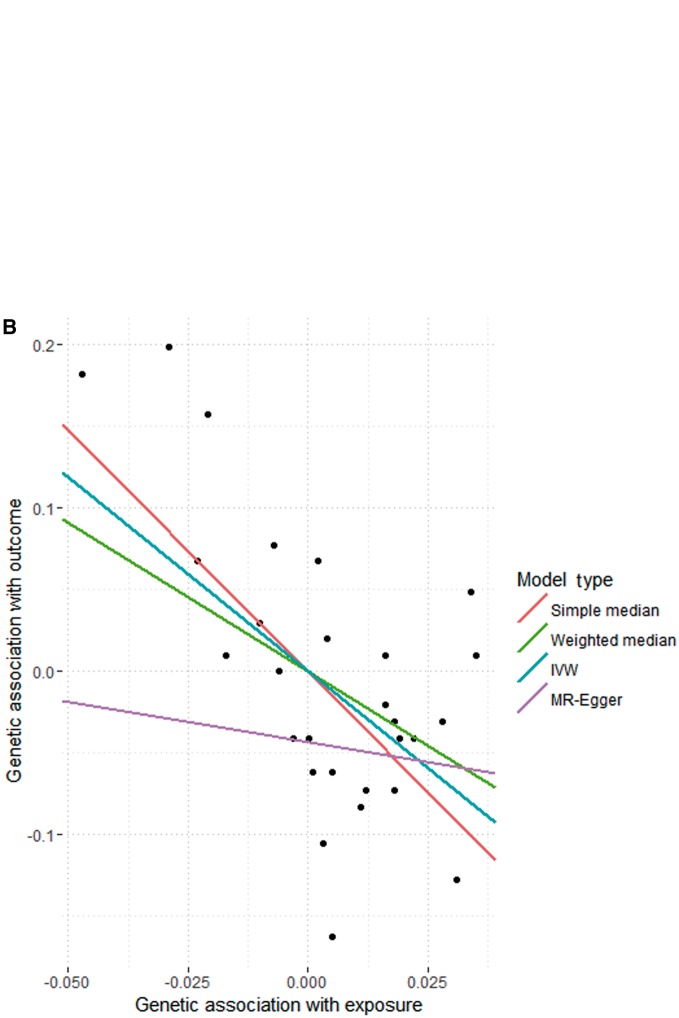
A. Code and output from implementation of various Mendelian randomization analysis methods using mr_allmethods() function for analysis of causal effect of high-density lipoprotein-cholesterol (HDL-c) on coronary heart disease (CHD) risk. B. Output from mr_plot() function applied to mr_allmethods() object. Static graph illustrating genetic associations with CHD risk (log odds ratios) against genetic associations with HDL-c (in standard deviation units). Lines represent causal estimates from the different methods. STD, standard.

This implements the IVW, MR-Egger, weighted and simple median methods, and puts the estimates, standard errors and confidence intervals into a table for easy comparison (Figure 1A).

There are two options for providing graphical output from this package. Both of these are implemented by the mr_plot() function. If the argument for the mr_plot() function is an MRAll object created using the mr_allmethods() function:

mr_plot(mr_allmethods(MRdata_HDL_CHD, method = ”main”))

then the result is a static graph illustrating the various causal estimates (Figure 1B). As can be seen in this example, the MR-Egger estimate differs substantially from the IVW and median-based estimates, suggesting some inconsistencies in the instrumental variable assumptions. Although the IVW method suggests that HDL-c is a causally protective risk factor for CHD, the MR-Egger estimate is compatible with the null, which is more in line with experimental findings.^19^

If the argument for the mr_plot() function is an MRInput object, for example:

mr_plot(MRdata_HDL_CHD)

then the result is an interactive graph of the genetic associations with the outcome against associations with the exposure. By default, error bars for the associations and the IVW estimate are also plotted. By mousing over a datapoint, an infobox appears with the name of the genetic variant and its associations with the exposure and with the outcome. This can be used for identifying genetic variants having heterogeneous associations with the outcome compared with other variants, and may be helpful in detecting variants to check for pleiotropy and possible violation of the instrumental variable assumptions.

**Figure 2:**
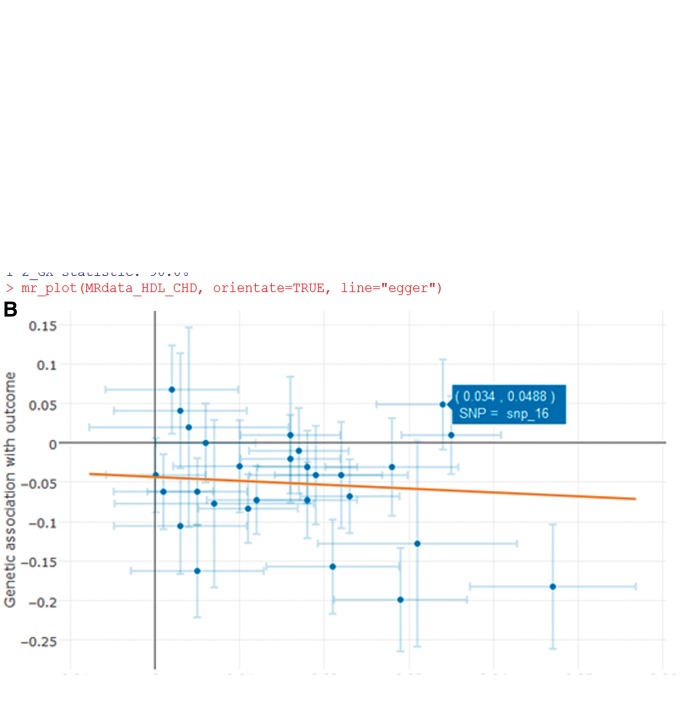
A. Code and output from implementation of MR-Egger method using mr_egger() function for analysis of causal effect of high-density lipoprotein-cholesterol (HDL-c) on coronary heart disease (CHD) risk. B. Output from mr_plot() function applied to mr_input() object. Screenshot of interactive graph illustrating genetic associations with CHD risk (log odds ratios) against genetic associations with HDL-c (in standard deviation units) with error bars representing 95% confidence intervals for the associations. The variants are all orientated to the HDL-c-increasing allele. The line represents the MR-Egger causal estimate. One of the genetic variants is highlighted by mousing over. The infobox gives the name of the variant (snp_16), and its associations with the exposure and with the outcome. STD, standard.

As an example, the output from the mr_egger() function applied to the HDL-c and CHD data provided as part of the package is presented in Figure 2A. The estimates of the slope and intercept parameters from the regression model are displayed, as well as their standard errors, 95% confidence intervals and associated *P*-values. The residual standard error from the weighted regression is substantially greater than 1, and the null hypothesis of the heterogeneity test is rejected, indicating that the genetic variants are not all estimating the same causal effect and suggesting the presence of pleiotropy for one or more variants. The I^2^ statistic quoted is a measure of weak instrument bias, and is described elsewhere.^20^ The mr_plot() command is then applied to the same data, and the MR-Egger estimate is illustrated using the code:

mr_plot(MRdata_HDL_CHD, orientate = TRUE, line = ”egger”)

A screenshot of the resulting interactive graph shows the possibility of identifying individual variants from the graph. The orientate = TRUE option ensures that all variants are plotted after orientation to the exposure-increasing allele, as this re-orientation is undertaken in the MR-Egger method.

Finally, although the primary method for inputting data into the package is directly by the user, it is possible to incorporate data from PhenoScanner, a database of genotype—phenotype associations that can be queried using a web browser.^16^ Two .csv files from PhenoScanner (v1.1, Little Miss Sunshine) are distributed as part of this package, representing the output from PhenoScanner for four genetic variants with proxies turned on and off. The user can then choose the exposure and outcome from those reported by PhenoScanner, and an MRInput object is created. For example:

path.proxies <- system.file(“extdata”, “vitD_snps_PhenoScanner_proxies.csv”, package = “MendelianRandomization”)

# this file has been downloaded from PhenoScanner (v1.1)

extract.pheno.csv(

exposure = “log(eGFR creatinine)”, pmidE = 26831199, ancestryE = “European”,

outcome = “Asthma”, pmidO = 20860503, ancestryO = “European”,

rsq.proxy = 0.6, file = path.proxies, snps = “all”)

This code takes the file provided by PhenoScanner, and outputs an MRInput object consisting of the genetic associations with the exposure “log(eGFR creatinine)” estimated in Europeans and reported by the study with PubMed ID 26831199,^21^ and genetic associations with the outcome “Asthma” estimated in Europeans and reported by the study with PubMed ID 20860503.^22^ If genetic associations for one or the other of the exposure and outcome are not reported for a particular variant, the function searches for a proxy variant with correlation measure *r^2^* greater than the threshold of 0.6 provided. Correlation estimates are provided by PhenoScanner, and can be calculated either for 1000 Genomes or HapMap participants of European descent. In this example, associations with asthma are not available for two variants; they are replaced by associations with suitable proxies. The output from the extract.pheno.csv() function can then be used in the mr_ivw() or similar functions for estimating a causal effect.

The eventual aim is to make this functionality seamless, so that PhenoScanner can be called directly from R as part of the MendelianRandomization package, or as a dependency to the package. Both this package and the PhenoScanner tool are still under development, and we hope that this aim will be achieved in the near future.

## Discussion

The MendelianRandomization package makes performing Mendelian randomization analyses using summarized data relatively straightforward. The package is still under development: future additions will include greater integration with the PhenoScanner and MR-Base [www.mr-base.org] platforms to enable increased automation of Mendelian randomization analyses, as well as a wider range of methods such as methods for multivariable Mendelian randomization.^23^ The package will also be updated as additional robust methods for performing Mendelian randomization using summarized data are developed.

As a final note of caution, the increasing automation of Mendelian randomization analyses is welcome for reducing the burden on investigators and the capacity for human errors to influence results, but there is a danger that the tools provided may facilitate large numbers of speculative Mendelian randomization analyses to be performed in an unprincipled way [see references 24–26 for some critical comments on Mendelian randomization]. It is important that Mendelian randomization is not performed in a way that avoids critical thought. In releasing this package, the hope is that it will lead to more comprehensive and more reproducible causal inferences from Mendelian randomization, and not simply add more noise to the literature.

## Supplementary Data

The appendix is available as Supplementary data at *IJE* online.

## Funding

Stephen Burgess is funded by a fellowship from the Wellcome Trust (grant number 100114).

## Supplementary Material

Supplementary Appendix
