# Supplementary material for: MendelianRandomization: an R package for performing Mendelian randomization analyses using summarized data
Source: Int J Epidemiol. 2017 Apr 7;46(6):1734–9. doi: 10.1093/ije/dyx034 (PMC5510723; doi:10.1093/ije/dyx034)
Supplement: Supplementary Appendix [file dyx034_supp.pdf]

## Appendix

The following is a series of commands that illustrates most of the range of the MendelianRandomization package aimed at causal or first-time users of the R software environment.

```
  # lines beginning with "#" are comments and are not run
install.packages("MendelianRandomization")
  # installation of the package is only necessary at first use

library(MendelianRandomization)

hdlc <-   c(0.002, 0.005, 0.003, 0.001, 0.011, 0.031, -0.003,
           -0.007, -0.021, 0.018, -0.017, -0.047, 0.022, -0.029,
            0.016, 0.034, 0.035, 0.019, 0.028, 0.0001, 0.016, 0.005,
           -0.01, -0.023,0.012, 0.018, -0.006, 0.004)
hdlcse <- c(0.004, 0.004, 0.004, 0.003, 0.003, 0.006, 0.004, 0.006,
            0.004, 0.003, 0.003, 0.005, 0.004, 0.004, 0.003, 0.004,
            0.003, 0.004, 0.004, 0.003, 0.003, 0.003, 0.004, 0.003,
            0.003, 0.003, 0.003, 0.006)
chdlodds   <- c(0.0677, -0.1625, -0.1054, -0.0619, -0.0834, -0.1278,
               -0.0408, 0.0770, 0.1570, -0.0305, 0.0100, 0.1823,
               -0.0408, 0.1989, 0.0100, 0.0488, 0.0100, -0.0408,
               -0.0305, -0.0408, -0.0202, -0.0619, 0.0296, 0.0677,
               -0.0726, -0.0726, 0.0000, 0.0198)
chdloddsse <- c(0.0286, 0.0300, 0.0310, 0.0243, 0.0222, 0.0667,
                0.0373, 0.0543, 0.0306, 0.0236, 0.0277, 0.0403,
                0.0344, 0.0335, 0.0378, 0.0292, 0.0253, 0.0319,
                0.0316, 0.0241, 0.0285, 0.0217, 0.0298, 0.0239,
                0.0220, 0.0246, 0.0255, 0.0647)
  # these data are provided as part of the package, but are
  # reproduced here to show how to enter data

mr_ivw(mr_input(bx=hdlc, bxse=hdlcse, by=chdlodds, byse=chdloddsse))
mr_ivw(mr_input(bx=hdlc, bxse=hdlcse, by=chdlodds,
  byse=chdloddsse))$Estimate
mr_ivw(mr_input(bx=hdlc, bxse=hdlcse, by=chdlodds, byse=chdloddsse),
  robust = TRUE)
mr_ivw(mr_input(bx=hdlc, bxse=hdlcse, by=chdlodds, byse=chdloddsse),
  penalized = TRUE)
mr_ivw(mr_input(bx=hdlc, bxse=hdlcse, by=chdlodds, byse=chdloddsse),
  robust = TRUE, penalized = TRUE)
mr_ivw(mr_input(bx=hdlc, bxse=hdlcse, by=chdlodds, byse=chdloddsse),
  model = "fixed", alpha = 0.01, distribution = "t-dist")

mr_egger(mr_input(bx=hdlc, bxse=hdlcse, by=chdlodds, byse=chdloddsse))
mr_egger(mr_input(bx=hdlc, bxse=hdlcse, by=chdlodds,
  byse=chdloddsse))$Estimate
mr_egger(mr_input(bx=hdlc, bxse=hdlcse, by=chdlodds, byse=chdloddsse),
  robust = TRUE)
mr_egger(mr_input(bx=hdlc, bxse=hdlcse, by=chdlodds, byse=chdloddsse),
  penalized = TRUE)
mr_egger(mr_input(bx=hdlc, bxse=hdlcse, by=chdlodds, byse=chdloddsse),
  robust = TRUE, penalized = TRUE)
mr_egger(mr_input(bx=hdlc, bxse=hdlcse, by=chdlodds, byse=chdloddsse),
  alpha = 0.01, distribution = "t-dist")

mr_median(mr_input(bx=hdlc, bxse=hdlcse, by=chdlodds, byse=chdloddsse))
mr_median(mr_input(bx=hdlc, bxse=hdlcse, by=chdlodds, byse=chdloddsse),
  weighting="weighted")
```

```
mr_median(mr_input(bx=hdlc, bxse=hdlcse, by=chdlodds, byse=chdloddsse),
  weighting="simple")
mr_median(mr_input(bx=hdlc, bxse=hdlcse, by=chdlodds, byse=chdloddsse),
  weighting="penalized")

mr_maxlik(mr_input(bx=hdlc, bxse=hdlcse, by=chdlodds, byse=chdloddsse))

mr_allmethods(mr_input(bx=hdlc, bxse=hdlcse, by=chdlodds, byse=chdloddsse),
  method="all")
mr_allmethods(mr_input(bx=hdlc, bxse=hdlcse, by=chdlodds, byse=chdloddsse),
  method="ivw")
mr_allmethods(mr_input(bx=hdlc, bxse=hdlcse, by=chdlodds, byse=chdloddsse),
  method="egger")
mr_allmethods(mr_input(bx=hdlc, bxse=hdlcse, by=chdlodds, byse=chdloddsse),
  method="median")

mr_plot(mr_input(bx=hdlc, bxse=hdlcse, by=chdlodds, byse=chdloddsse),
  line="ivw")
mr_plot(mr_input(bx=hdlc, bxse=hdlcse, by=chdlodds, byse=chdloddsse),
  line="ivw", interactive = FALSE)
mr_plot(mr_input(bx=hdlc, bxse=hdlcse, by=chdlodds, byse=chdloddsse),
  line="ivw", interactive = FALSE, labels=TRUE)
mr_plot(mr_input(bx=hdlc, bxse=hdlcse, by=chdlodds, byse=chdloddsse),
  error=FALSE, line="egger", orientate = TRUE)

mr_plot(mr_allmethods(mr_input(bx=hdlc, bxse=hdlcse,
  by=chdlodds, byse=chdloddsse), method="all"))
mr_plot(mr_allmethods(mr_input(bx=hdlc, bxse=hdlcse,
  by=chdlodds, byse=chdloddsse), method="ivw"))
mr_plot(mr_allmethods(mr_input(bx=hdlc, bxse=hdlcse,
  by=chdlodds, byse=chdloddsse), method="egger"))
mr_plot(mr_allmethods(mr_input(bx=hdlc, bxse=hdlcse,
  by=chdlodds, byse=chdloddsse), method="median"))

path.noproxy <- system.file("extdata",
  "vitD_snps_PhenoScanner.csv",
  package = "MendelianRandomization")
path.proxies <- system.file("extdata",
  "vitD_snps_PhenoScanner_proxies.csv",
  package = "MendelianRandomization")
 # these two files from PhenoScanner are provided
 # as part of the MendelianRandomization package

extract.pheno.csv(
  exposure = "log(eGFR creatinine)", pmidE = 26831199,
  ancestryE = "European",
  outcome = "Tanner stage", pmidO = 24770850, ancestryO = "European",
  file = path.noproxy)

extract.pheno.csv(
  exposure = "log(eGFR creatinine)", pmidE = 26831199,
  ancestryE = "European",
  outcome = "Tanner stage", pmidO = 24770850, ancestryO = "European",
  rsq.proxy = 0.6, file = path.proxies)

extract.pheno.csv(
  exposure = "log(eGFR creatinine)", pmidE = 26831199,
  ancestryE = "European",
  outcome = "Asthma", pmidO = 20860503, ancestryO = "European",
  rsq.proxy = 0.6, file = path.proxies)
```
